# Clinicians’ Perceptions of the Appropriateness of Neurocritical Care for Patients with Spontaneous Intracerebral Hemorrhage (ICH): A Qualitative Study

**DOI:** 10.1007/s12028-020-01145-5

**Published:** 2020-12-02

**Authors:** Siobhan Mc Lernon, David Werring, Louise Terry

**Affiliations:** 1 School of Health and Social Care, London South Bank University, 103 Borough Road, London, SE1 OAA UK; 2 Stroke Research Centre, UCL Institute of Neurology, First Floor, Russell Square House, 10-12 Russell Square, London, WC1B 5EH UK

**Keywords:** Intracerebral hemorrhage (ICH), Prognostic uncertainty, Neurocritical care (NCC), Perceived inappropriate care (PIC), Distress, Doctors, Nurses

## Abstract

**Background and Objective:**

Clinicians working in intensive care frequently report perceptions of inappropriate care (PIC) situations. Intracerebral haemorrhage (ICH) is associated with high rates of mortality and morbidity. Prognosticating after ICH is complex and may be influenced by clinicians’ subjective impressions and biases, which may, in turn, influence decision making regarding the level of care provided. The aim of this study was to qualitatively explore perceptions of neurocritical care in relation to the expected functional outcome for ICH patients.

**Design:**

Qualitative study using semi-structured interviews with neurocritical care doctors and nurses.

**Setting:**

Neurocritical care (NCC) department in a UK neuroscience tertiary referral center.

**Subjects:**

Eleven neurocritical care nurses, five consultant neurointensivists, two stroke physicians, three neurosurgeons.

**Intervention:**

None.

**Measurements and Main Results:**

We conducted 21 semi-structured interviews and identified five key themes: (1) prognostic uncertainty (2) subjectivity of good versus poor outcome (3) perceived inappropriate care (PIC) situations (including for frail elderly patients) (4) challenging nature of decision-making (5) clinician distress.

**Conclusions:**

Caring for severely affected ICH patients in need of neurocritical care is challenging, particularly with frail elderly patients. Awareness of the challenges could facilitate interventions to improve decision-making for this group of stroke patients and their families, as well as measures to reduce the distress on clinicians who care for this patient group. Our findings highlight the need for effective interdisciplinary shared decision making involving the family, taking into account patients’ previously expressed values and preferences and incorporating these into bespoke care planning.

## Introduction

Spontaneous intracerebral hemorrhage (ICH) is the deadliest, least-treatable stroke type: case fatality at 1 month is 30-40%, and only 20% of survivors regain independence [1]. ICH mainly affects older adults, often those with high blood pressure (BP) and other vascular comorbidities.

Data suggest that the poor outcomes associated with ICH frequently lead doctors and nurses to believe that on-going treatment is of little benefit; this pessimistic viewpoint, particularly regarding those perceived as “frail” on admission, results in the early withholding or withdrawal of treatment in patients, some of whom might have had an acceptable outcome if treated actively [2, 3].

ICH guidelines now recommend avoidance of early withholding or withdrawal of treatment and transfer to higher-level care settings for those that require it [4]. Rapid lowering of BP and supporting those with low levels of consciousness optimizes functional recovery [5]. Consequently, growing numbers of stroke patients are admitted to intensive care for supportive management. This, combined with a concomitant growing incidence of elderly patients suffering a stroke, may account for increased rates of interventions in patients at high risk of poor outcomes [6]. However, the elderly have high mortality rates after ICH and prolonged lengths of stay in intensive care [7, 8]. Mortality rates are also higher in frail compared with non-frail patients [8].

Advances in the availability and feasibility of life-supporting technologies for stroke patients and limitations in predicting mortality and morbidity after ICH bring new urgency to understanding the appropriateness of neurocritical care for ICH patients. Intensive care clinicians frequently report perceptions of inappropriate care (PIC) [9, 10]. This is thus an important area for research in light of associated poor ICH outcomes after intensive care [6, 11]. As part of a mixed methods doctoral study, we aimed to explore clinicians’ perceptions of the appropriateness and outcome after neurocritical care (NCC) for ICH patients.

## Methods

Qualitative semi-structured interviews were conducted. An interview schedule asking a series of open-ended questions (Supplemental Digital Content) was developed based on the literature review, expert opinion and clinical experience [12]. Face validity was confirmed by experienced researchers, and clinical experts. The chief investigator (SML) conducted one-to-one semi-structured interviews, approximately 30 min each, between September 2018 and March 2019. Interviews were audio-recorded, transcribed verbatim, and anonymized. Standards for Reporting Qualitative Research (SRQR) were adhered to [13].

### Sample and Setting

#### Sample

Participants were recruited from within a UK Neurocritical Care (NCC) Department in a neuroscience tertiary referral center in a multi-site acute teaching NHS hospital Trust within a large metropolitan area in the South East of England. Participants were recruited via email invitation using distribution lists. A purposive sample was used as we sought accessible and consenting participants with various job roles, experience and clinical grade so that a range of opinions and perspectives were captured [14].

Inclusion criteria were: nurses and doctors with different levels of responsibilities and over 6 months’ experience of working within the neurosurgical critical care unit, which is within the NCC department. This was to ensure that participants had experience of caring for ICH patients. Agency nurses and locum physicians were excluded. There were no difficulties with recruitment and interviews were stopped once data saturation was reached (i.e., no new information or themes were observed in the data) [15].

#### Setting

The neurosurgical critical care unit has nine level three beds with a nurse to patient ratio of 1:1 and a high dependency unit (HDU) which has ten level two beds with a 2:1 nurse to patient ratio. At the time of data collection, there were in the region of 80 nursing personnel with a variety of clinical grades and experience working within the neurosurgical critical care. The neurosurgical critical care is anesthetic led by seven consultant neurointensivists and rotating junior physicians. Over the last three consecutive years, the neurosurgical critical care received approximately 100-110 ICH patients (average 9 per month) per year and is considered to be a high-volume center for stroke care. This ensured that the study setting was representative of the ICH population of patients and the participants under study could meet the desired study aims.

Patients with ICH are routinely admitted to the neurosurgical critical care as per protocol via two pathways: 1: The stroke pathway 2: The neurosurgical pathway. Neurosurgical and stroke input is available for all patients as appropriate. ICH patients receive neurosurgery based on clinical need and following discussion between the attending neurointensivist, stroke consultant and neurosurgeon. EVD insertion is not routine for intraventricular hemorrhage (IVH) with hydrocephalus but is based on clinicoradiological characteristics and clinical need. From 2017 to 2019, 47/306 (15%) of ICH patients admitted to neurosurgical critical care were dead on discharge. Off those dead, nine (19%) had brainstem testing (BST) within 1–2 days of admission, and six (13%) had treatment withdrawn within 1–3 days of admission. The remainder died due to neurological and/or physiological deterioration.

### Analysis

Reflexive thematic analysis (TA) [16] was used for analysis to identify patterns across the dataset in relation to the research aim [17]. The inductive, iterative nature of thematic analysis enabled the exploration of perceptions surrounding caring for a particular stroke group, which is currently under-researched with no clear theory from which to derive a framework for deduction. The inductive, iterative nature of thematic analysis revealed the “nuanced reality” of participants’ experiences. Interviewing stopped when no more themes emerged (i.e., data saturation). Table 1 describes the strategies that were used to increase trustworthiness and rigor [18]. Ethical approval was granted by the Heath Research Authority (HRA) in May 2018 and ratified by London South Bank University Research Ethics Committee in September 2018. Informed written consent was obtained from participants.

**Table 1:** Trustworthiness strategies [18]

Phase	Process	Means of establishing trustworthiness through each phase of analysis
1: Detailed reading of the transcripts, to develop familiarization and full immersion with the data	This involved “repeated reading” searching for meanings and patterns. Noting down initial ideas	Prolonged engagement with the data and documentation of all initial thoughts in field notes
2: Generating initial codes	Interesting features of the data were coded in a systematic way across the data set, collating data relevant to each code using an excel spread sheet	Peer debriefing in which the initial codes were discussed with experienced qualitative researchers. A reflexive journal was used to document decisions and help to synthesize the data. To assess inter-coder reliability, four transcripts were independently coded by an experienced researcher for any discrepancies
3: Searching for themes	Codes were collated into potential themes, gathering all relevant data to each potential theme. Initial ideas of themes were outlined on paper flip charts to develop a visual representation of the data	Use of diagrams on paper charts to make sense of theme connections and hierarchies of concepts. Use of photographs to show how themes were developed
4: Reviewing themes	The themes were then checked to see if they worked in relation to the coded extracts (level 1) and the entire dataset (level 2). Following this, a thematic map of the analysis was generated on paper flip charts	Themes and subthemes were discussed and reviewed at regular intervals with the research team. To test for representiveness of themes, the researcher returned frequently to the raw data at various stages
5: Defining and naming themes	On-going refinement of the themes was undertaken to portray the overall story that the analysis revealed, generating clear definitions and names for each theme	Peer debriefing and clear documentation of meetings regarding theme development and select representative statements
6: Producing the report	A selection of vivid, compelling extract samples were included in the final analysis relating back to the research aim and literature	Member checking by going back to several participants and checking that themes reflected the transcripts and peer debriefing. Clear description of decision-making and analytical process

## Results

**Fig. 1:**
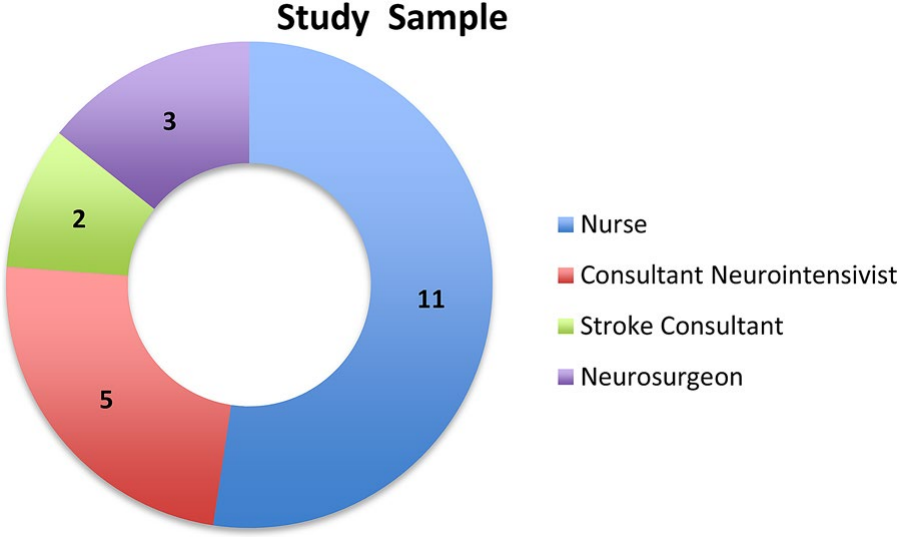
Study sample

Figure 1 shows the staff groups comprising the study sample. The size of the 21 participants were included in the sample was based on the number and skill mix at the time of data collection. The following is a breakdown of those that participated: 11/80 nurses, 5/7 consultant neurointensivists, 2/5 vascular neurosurgeons and 2/4 stroke consultants; no junior anesthetists or senior house officers took part.

Interviews had a duration of 12–30 min (Total 397 min). The majority of participants had > 5 years’ experience of caring for neurocritically ill patients. Table 2 describes the job role, grade, and experience of participants including nurses with neuroscience training.

**Table 2:** Participants job role, position and grade, experience years, neuroscience nurse training Y (Yes) N (No) yrs (Years)

Job role	Position and grade	Length of neurocritical care experience (Yrs)	Nurses trained in neuroscience Y/N
Nurse	Band 6	5	Y
Nurse	Band 7	10	Y
Nurse	Band 6	13	Y
Nurse	Band 7	15	Y
Doctor	Consultant Neurointensivist	10	–
Nurse	Band 5	1	N
Nurse	Band 6	5	Y
Doctor	Consultant Neurointensivist	23	–
Doctor	Consultant Neurointensivist	17	Y
Nurse	Band 7	13	Y
Doctor	Consultant Neurointensivist	4.5	–
ClinicalNurseSpecialist(CNS)	Band 7	25	Y
Nurse	Band 5	2	N
Doctor	Stroke Consultant	10	–
Doctor	Stroke Consultant	1	–
Doctor	Consultant Neurointensivist	15	–
Nurse	Band 6	3	Y
Doctor	Consultant Neurosurgeon	12	–
Nurse	Band 7	15	Y
Doctor	Consultant Neurosurgeon	20	–
Doctor	Neurosurgical Senior Registrar	8	–

**Fig. 2:**
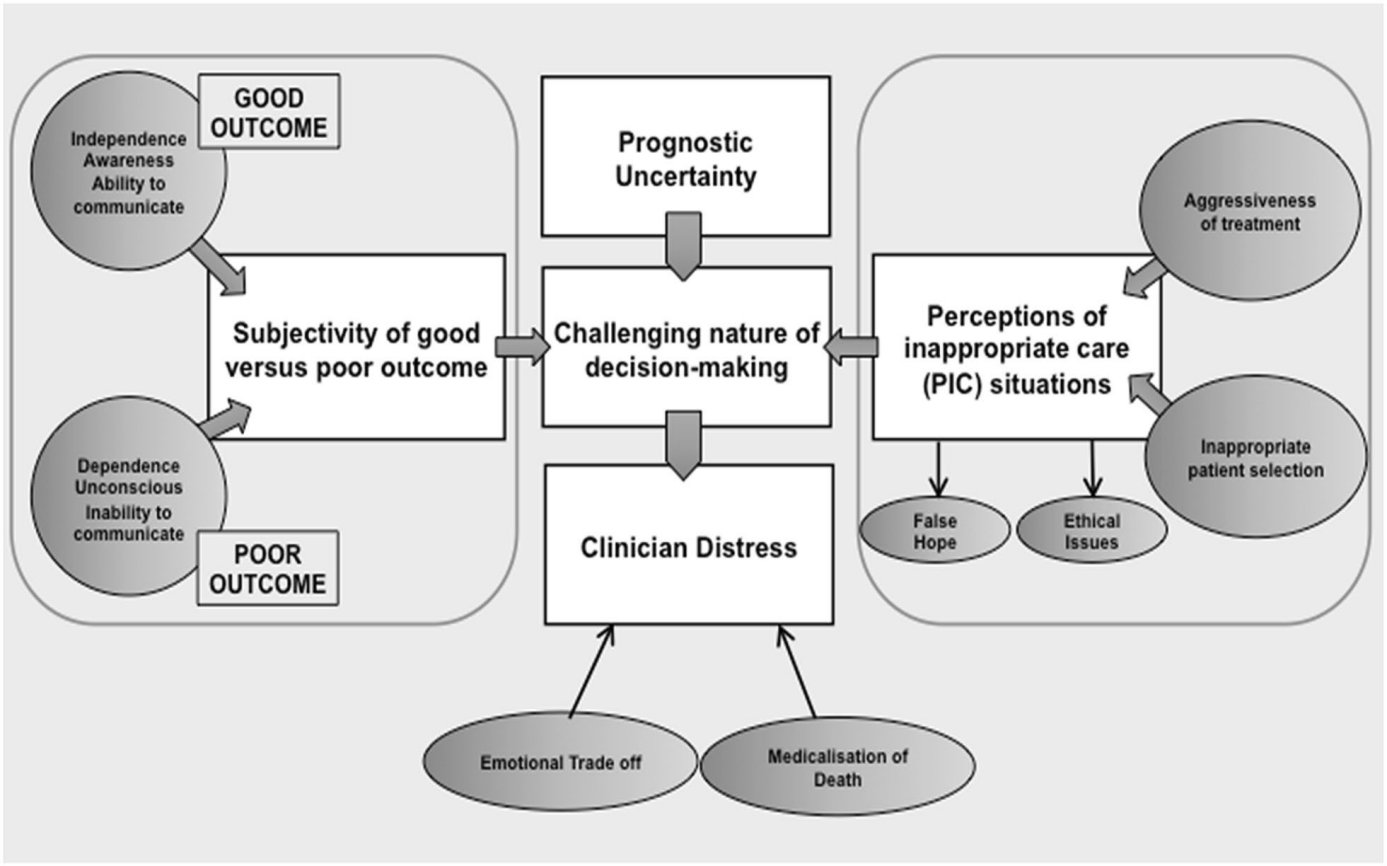
Themes and subthemes

Five major themes were identified from the 21 interviews conducted (11 neurocritical care nurses, five consultant neurointensivists, two stroke physicians, three neurosurgeons). Each is presented in turn below with exemplar quotes. Figure 2 summarizes the main themes and subthemes.

### Prognostic uncertainty

Clinicians found ICH patients to be a challenging stroke group due to an inability to predict outcome as some patients may be in a deep coma before showing signs of recovery.

We have had patients who have had some very deep bleeds who have then started obeying 2 or 3 weeks later because they are a very slow group to do anything. (N1)

Neurosurgeons questioned the benefits of neurosurgery.

There is no agreement [on] what is the best way of doing things. On one extreme, people do very aggressive surgery, others who do no surgery at all. (S1)

Patient characteristics known to be predictive of poor outcome such as older age, existing comorbidities, and frailty presented challenges.

…anyone who’s in their 80’s and you can see that they are frail, they’ve got poor muscle mass and they are thin and they’ve got other comorbidities, it is obvious they are not going to do very well. (D7)

Neuroimaging findings such as the size and location of ICH were considered predictive of patient outcome but even those were unreliable.

They can have a really bad scan but they look different when you assess them GCS-wise…it’s looking at the GCS really and how they are. (N11)

Some ICH patients recovered much better than initially expected:

… you see these patients as pretty all doom and gloom and he made me realise that with good supportive care these patients can do very well. (D7)

Prognostic uncertainty was compounded by uncertainty about long-term outcome. Participants considered knowing long-term outcomes would help develop their ability to prognosticate and advise families.

The challenges are predicting outcome really. It’s knowing what’s going to happen to them, a very hard thing to predict to families. (D2)

Some participants wanted to know what survival meant from the patients’ perspective.

… is he still glad to be alive or is he deeply upset that he can’t do the things that he used to? (D4)

### Subjectivity of Good Versus Poor Outcome

Two domains were identified as important in terms of outcome: cognition and functionality. Participants perceived survivors who were aware and able to interact with their surroundings as a good outcome.

Cognition is the most important. (S3)

Independence and ability to conduct activities of daily living (ADLs), was considered a good outcome.

… to function again…like every other person. The relatives want their dad back, they don’t want their dad to be back with a disability, so a good outcome would be to recover from this illness as fully as possible. (N2)

Survival at the cost of severe disability requiring full care with ADLs or in a persistent unconsciousness state was consistently considered a poor outcome.

…the worst ones are the ones who are left unable to communicate, unable to look after themselves with no sort of way out, I think. (D2)

Although death was generally considered a poor outcome, one participant judged a 97-year old ICH patient’s death differently.

Sometimes a good outcome is death, cos it’s natural, isn’t it? (N3)

Participants recognised that perceptions of outcome were subjectively interpreted by individual clinicians, individual patients and their families.

…I always try to remember not to judge my perception of what a good quality of life is by others and remember that people’s idea of quality of life changes down the line as well…it’s fluid. (N5)

Pessimism was evident.

They never really return to much independence and they, very often, have a pretty miserable time in hospital afterwards and end up dying from medical complications. (D7)

However, clinician’s perceptions could change.

…a patient who looked like they were going to do very badly indeed…he was very happy with his outcome of being completely hemiplegic and happy that he had lived and I suppose that was an important lesson for me. (D4)

### Perceived Inappropriate Care (PIC) Situations

Some patients were inappropriately selected for admission to neurocritical care for on-going care based on clinical data.

Pupils had already blown…it wasn’t fair on the family to have to come to another hospital and give them hope and things. (N1)

…a patient who is elderly with lots of comorbidities with a big clot, what is the point? (D2)

Inappropriate admissions included frail patients with pre-existing comorbidities and already fully dependent on others for ADL.

Looking at their past medical history, were they, very, very dependent on carers and things like that?…you can already see the failure. (N8)

Participants frequently described situations where the aggressiveness of on-going treatment seemed inappropriate.

… they gave her decompressive craniectomies, which was absolutely horrendous, and I really could not believe why we were treating this lady.(N3)

Prolonged use of aggressive supportive treatment was perceived as ‘cruel’.

When they need more blood pressure control and they start getting a chest infection and everything else, it starts getting cruel, more cruel than kind.(N1)

Admitting severely affected ICH patients to neurocritical care gave “false hope” to families making their expectations unrealistic and challenging to manage.

…then you are in a situation where the family are expecting a miracle because they are still waiting for them to wake up. They have been given false hope. (D2)

PIC situations raised ethical issues surrounding futility, best interests and the use of resources.

Sometimes you feel things are a bit futile…you are doing things that are…not in the patient’s best interests. (N3)

Death was frequently perceived as being ‘medicalised’.

Perhaps the person just has not been allowed to die from an end-of-life event. We are intervening inappropriately to prolong a dying process. (N8)

### Challenging Nature of Decision-Making

Clinicians found decision-making throughout each stage of the patient’s journey challenging: patient selection in A&E, whether to perform neurosurgery, the duration and aggressiveness of treatment, and when to transition to palliative care. Selecting patients for admission to neurocritical care was difficult due to a lack of information regarding health and dependence levels.

Lack of holistic assessment. (D6)

Knowing patient’s wishes in advance aided the decision-making process surrounding the aggressiveness and duration of on-going treatment.

…if you know someone’s views very early on, it can make difficult decisions… a little easier. (N4)

Participants were alert to avoiding early-phase pessimism.

You shouldn’t have a preconception really early on in the decision process of what their outcome is going to be, no matter what their age. (S2)

There was consensus that most ICH patients should be given a chance of early supportive care.

Throw the kitchen sink at everyone.” (D5)

Participants considered that decisions about further supportive treatment should wait until ICH patients had been given a trial of treatment in neurocritical care for at least 48-72 h. Several spoke about putting limits on the level of treatment given.

Agreeing a ceiling of care, in my opinion, is ethically acceptable. Saying that if the patient has a bad chest infection maybe he should not be on a ventilator again. (S1)

Deciding to transition to palliative care was particularly difficult for the direct care team and families.

I find this really difficult because it’s really hard to get my head around. So I should imagine families must find it really hard to get their heads around. (N5)

One participant talked about the need for more shared decision-making.

We are not good at meeting together and exploring all of these points of view together and sharing the responsibility for continuing in an active way or moving to palliative care. (D6)

### Clinician distress

Participants described ICH patients as ‘complex’ due to the challenges they present:

Complex group, with medical complexities that are unfamiliar and challenging. (D6)

Participants talked emotively about feelings of distress, sadness, and guilt.

Excessive treatment…that we perceived not…to be worthwhile or sensible to put the patient through or put the family through that degree of torture in ITU. (D3)

Nurses frequently expressed sadness when patients survived with severe disability.

Patients that don’t wake up, low GCS, extending, they make me feel quite sad. (N1)

Nurses particularly felt conflicted.

When patients have a very, very extensive bleed and we know that even if they survive they will be severely disabled and we still admit them and give them full treatment to confirm that after 4,5,6 weeks that they are doing very poorly because of the extent of the bleed, so is it worth it? (N9)

The medicalisation of death caused distress.

When you can see that actually we are doing more harm than good here by just prolonging the inevitable. (N10)

Clinicians offset feelings of distress by providing good palliative care.

We fight for people…to transition them to palliative care…I wouldn’t say it is a failure on our part, I think we should view it as another process to aid the patient in a different way. (N10)

We can actually give them the proper palliative care. (N7)

## Discussion

Participants found clinical decision-making challenging, partly because of prognostic uncertainty. Accurately prognosticating after ICH is complex, decisions may be influenced by subjective impressions and biases [19, 20]. Overly optimistic prognostication and over-treatment can cause excessive suffering, burden and cost, yet premature withdrawal of treatment can result in patients dying who might otherwise have had acceptable outcomes with appropriate treatment [19]. Participants in our study were trying to balance fears of over-treatment against premature treatment withdrawal since those in the worst prognostic category sometimes make a meaningful recovery [4]. Severe brain injury (including ICH) often takes many months to achieve maximal functional recovery [22]; assessment of outcome at discharge or 6 months may underestimate the quality of longer-term survival [23].

Participants acknowledged the subjective nature of outcome and difficulty separating their personal views on outcome from those of the patient/family. This made it difficult to advise if outcomes would be “good” or “poor.” The issue of perceived outcome and the “paradox” whereby outcome is considered good from the views of the clinician, as opposed to the patient’s family or carers, will always remain a “gray area” of practice in neurocritical care especially when we consider whether mRS scores of 3 or 4 can be considered “good”.

Clinicians thus need to obtain an understanding of what aspects of recovery are the most important aspects of “good” recovery to the individual patient [21]. However, shared decision-making [24] can be challenging after ICH because of prognostic uncertainty, patients who are too unwell to express views, and families and carers not knowing the patient’s wishes. Moreover, societal expectations and patients’ views of what a “life worth living” actually is are constantly being recalibrated; many patients who are severely disabled appear to adapt to their situation (response shift) and report higher quality of life than anticipated [24]. This so-called “disability paradox” adds to the challenge of decision-making after ICH [25].

Participants found decision-making surrounding transition to palliative care difficult. Guidelines for end-of-life care advocate collaboration [21]. The DISPROPRICAS study, which examined perceptions of excessive care by clinicians, concluded that ethically based clinical decision making has to rely on both individual perceptions and objective criteria, followed by interdisciplinary discussions that enrich the process for the benefits of the patient and their family [26].

Therapeutic nihilism has historically pervaded the management of ICH with non-resuscitation orders frequently used early on, which increase mortality even after adjustment for ICH severity [3, 27]. Participants expressed pessimistic viewpoints based on objective ICH patient characteristics on admission colored by their subjective views regarding outcome. These findings concur with evidence that suggests that therapeutic nihilism regarding ICH persists in UK stroke practice [28]. Guidelines recommend that after ICH onset a therapeutic trial of early aggressive care should be initiated, and new non-resuscitation orders postponed until at least the second day of hospitalization [4]. Resuscitation status should not limit aggressive, guideline-directed therapy unless otherwise explicitly indicated. Guidelines, in combination with studies that have shown better outcomes than previously thought [4, 29], have consequently led to more initial intensive care for ICH patients in critical care settings. The NCC in which our study was conducted utilizes nationally based hospital protocols regarding organ donation when appropriate. However, the duty to preserve life means prioritizing the ICH patient and not a potential organ recipient. For these reasons, clinicians are encouraged to practice self-awareness strategies and to identify previously held biases and emotional states while caring for stroke patients [30, 31]. This may limit therapeutic nihilism in clinical practice.

PIC situations centered on the intensity of treatment which raised ethical issues surrounding futility, the inappropriate use of resources, and giving families false hope. Our findings concur with other studies, which have identified prognostic uncertainty, treatments that led to more suffering and dependence, and inappropriate use of resources [9, 10, 31–33].. Irrespective of PIC situations, participants unanimously believed that most ICH patients should receive a time-sensitive trial of supportive aggressive management in NCC during the acute phase. These findings reflect current recommendations for the management of perceived devastating brain injury after hospital admission [34].

Our study clearly reveals the presence of clinician distress while treating ICH patients in NCC. Participants feared saving life at the expense of long-term patient suffering. Treating severely affected, elderly frail ICH patients with existing comorbidities was perceived as fraught with ethical dilemmas. As older age and pre–ICU frailty is associated with increased mortality [8, 35], it seems logical that increased use of frailty risk scores [36] in the elderly may help to identify those ICH patients who, on admission, are at greater risk of adverse outcomes after treatment in intensive care. It is possible that the distress expressed by clinicians could be alleviated if more long-term clinical outcomes and quality of life assessments were available. This may facilitate more accurate prognostication and help limit therapeutic nihilism.

Providing the patient with a dignified death appeared therapeutic and perhaps provided an *“emotional trade*-*off”,* helping participants to manage distress at not being able to save the patient’s life. The commencement of palliative care in neurocritical care is an important part of stroke care and management. Addressing the palliative care needs of severely affected ICH patients and families can improve the quality of life of stroke patients, their families, and their care providers [21].

## Strengths and Limitations

This study has strengths. Data were obtained from highly experienced clinicians working in a large UK neuroscience tertiary referral center considered to be a high-volume center representative of ICH patient care. This therefore offers a unique and original insight into a group of clinicians’ perceptions with regard to the appropriateness of neurocritical care for ICH patients. This, we believe will be of interest to the neurocritical care community as it sheds light on the many challenges posed by the management of severely affected stroke patients.

However, there are also some limitations. This was a single center study, which limits the generalizability of the findings. Future research should include more than one NCC setting to explore a wider range of clinicians’ perceptions and to make comparisons. This present study has sought qualitative insights but survey-based research with structured questions and objective assessment could be useful to provide further insight.

## Conclusion and Recommendations

Caring for severely affected ICH patients in NCC is challenging due to prognostic uncertainty, subjective perceptions of outcome, and PIC situations. These factors impact on the decision-making process and can result in clinician distress. More feedback to clinicians who care for ICH patients in critical care regarding clinical outcomes and quality of life data from both the survivors and carers perspective may help to alleviate clinician distress as well as limit therapeutic nihilism while caring for this patient group. A more holistic assessment in the emergency department, perhaps combined with an increased use of frailty risk scores on admission, might assist in the decision-making process surrounding ICH patient selection for neurointensive care. Our findings highlight the need for effective interdisciplinary shared decision-making involving the family, taking into account patients’ previously expressed values and preferences and incorporating these into bespoke care planning.

## References

[1] Van Asch CJ, Luitse MJ, Rinkel GL (2010). Incidence, case fatality, and functional outcome of intracerebral haemorrhage over time, according to age, sex, and ethnic origin: a systematic review and meta-analysis. Lancet Neurol.

[2] Beaker KJ, Baxter AB, Cohen WA (2001). Withdrawal of support in intracerebral hemorrage may lead to self-fulfilling prophecies. Neurology.

[3] Zahuranec DB, Brown DL, Lisabeth LD (2007). Early care limitations independently predict mortality after intracerebral haemorrhage. Neurology.

[4] Hemphill JC, Greenberg SM, Anderson CS (2015). Guidelines for the management of spontaneous intracerebral hemorrhage. A Guideline for Healthcare Professionals From The American Heart Association and American Stroke Association. Stroke.

[5] Royal College of Physicians (RCP) National clinical guideline for stroke. Prepared by the Intercollegiate Stroke Working Party, 5th Edition. Royal College of Physicians. 2016. London.

[6] Alonso A, Ebert AD, Kern R, Rapp S (2015). Outcome predictors of acute stroke patients in need of intensive care treatment. Cerebrovasc Dis.

[7] Heyland D, Cook D, Bagshaw SM (2015). The very elderly admitted to ICU: a quality finish?. Crit Care Med.

[8] Hope AA, Gong MN, Guerra C, Wunsch H (2015). Frailty before critical illness and mortality for elderly medicare beneficiaries. J Am Geriatr Soc.

[9] Piers RD, Azouley E, Ricou B (2011). Perceptions of appropriateness of care among european and israeli intensive care unit nurses and physicians. JAMA.

[10] Piers RD, Azouley E, Ricou B (2014). Inappropriate care in European ICU’s: confronting views from nurses and junior and senior physicians. Chest.

[11] Wartenberg KE, Wang X, Munoz-Venturelli P (2016). Intensive care unit admission for patients in the INTERACT2 ICH blood pressure treatment trial: characteristics, predictors, and outcomes. Neurocrit Care.

[12] Onwuegbuzie AJ, Leech NL (2007). Validity and qualitative research: an oxymoron?. Qual Quant.

[13] O’Brien BC, Harris IB, Beckman TJ (2014). Standards for reporting qualitative research: a synthesis of recommendations. Acad Med.

[14] Bryman A (2016). Social research methods.

[15] Saunders B, Sim J, Kingstone T (2018). Saturation in qualitative research: exploring its conceptualization and operationalization. Qual Quant.

[16] Braun V, Clarke V (2006). Using thematic analysis in psychology. Qual Res Psychol.

[17] Braun V, Clarke V (2013). Successful qualitative research: a practical guide for beginners.

[18] Lorelli S, Nowell JM, Norris DE (2017). Thematic analysis: striving to meet the trustworthiness criteria. Int J Qual Methods.

[19] Zahuranec DB, Fagerlin A, Sanchez BN (2016). Variability in physician prognosis and recommendations after intracerebral haemorrhage. Neurology.

[20] Caulfield AF, Gabler L, Eyngorn I (2010). Outcome prediction in mechanically ventilated neurologic patients by junior neurointensivists. Neurology.

[21] Holloway RG, Arnold RM, Creutzfeldt C (2014). Palliative and end-of-life care in stroke. A Statement for Healthcare Professionals from the American Heart Association/American Stroke AssociationA Statement for Healthcare Professionals from the American Heart Association/American Stroke Association. Stroke.

[22] Kyoung BL, Seong HL, Kyung HK (2015). Six-month functional recovery of stroke patients: a multi-time-point study. Int J Rehabil Res.

[23] Visvanathan A, Mead G, Dennis M (2019). Maintaining hope after a disabling stroke: a longitudinal qualitative study of patients experiences, views, information needs and approaches towards making treatment decisions. PLoS ONE.

[24] Visvanathan A, Mead G, Dennis M (2017). Shared decision making after severe stroke—how can we improve patient and family involvement in treatment decisions?. Int J Stroke.

[25] Albrecht GL, Devlieger PJ (1999). The disability paradox: high quality of life against all odds. Soc Sci Med.

[26] Benoit DD, Jensen HI, Malmgren J (2018). Outcome in patients perceived as receiving excessive care across different ethical climate: a prospective study in 68 intensive care units in Europe and the USA. Intensive Care Med.

[27] Hemphill JC, White DB (2009). Clinical Nihilism in neuroemergencies. Emerg Med Clin N Am.

[28] Parry-Jones AR, Paley L, Bray BD (2016). On behalf of the SSNAP Collaborative Group. Care limiting decisions in acute stroke and association with survival: analyses of UK national quality register data. J Stroke.

[29] Sattur MG, Spiotta AM (2020). Commentary: efficacy and Safety of Minimally Invasive Surgery With Thrombolysis in Intracerebral Haemorrhage Evacuation (MISTIE III): a randomized, controlled, open-label, blinded endpoint phase 3 trial. Neurosurgery.

[30] Creutzfeldt CJ, Holloway RG (2012). Treatment decisions after severe stroke: uncertainty and biases. Stroke.

[31] Palda VA, Bowman KW, McLean RF, Martin G, Chapman BM (2005). “Futile” care: do we provide it? Why? A semi-structured, Canada–wide survey of intensive care unit doctors and nurses. J Crit Care.

[32] Mobley MJ, Rady MY, Verheijde JL (2007). The relationship between moral distress and perception of futile care in the critical care unit. Intensive Crit Care Nurs.

[33] Sibbald R, Downar J, Hawryluck L (2007). Perceptions of futile care among caregivers in intensive care units. CMAJ.

[34] Faculty of Intensive Care Medicine: A Consensus Statement: Management of Perceived Devastating Brain injury after hospital admission. 2018. UK. 10.1016/j.bja.2017.10.002 29397121

[35] Radholm K, Arima H, Lindley RI (2015). Older age is a strong predictor for poor outcome in intracerebral haemorrhage: the INTERACT 2 study. Age Ageing.

[36] Gilbert T, Neuburger J, Kraindler J (2018). Development and validation of a Hospital Frailty Risk Score focusing on older people in acute care settings using electronic hospital records: an observational study. Lancet.

